# A Silenciosa Transição da Menopausa: A Interseção entre Glândulas Salivares, Condição da Cavidade Oral e Saúde Cardiovascular

**DOI:** 10.36660/abc.20250737

**Published:** 2026-05-18

**Authors:** Silvia Vanessa Lourenço, Giovanna Piacenza Florezi, Cibele Pelissari, Flavio Carneiro Hojaij, José Pinhata Otoch, José Antonio Franchini Ramires

**Affiliations:** 1 Universidade de São Paulo Faculdade de Medicina Departamento de Cirurgia São Paulo SP Brasil Departamento de Cirurgia, Faculdade de Medicina, Universidade de São Paulo, São Paulo, SP – Brasil; 2 Universidade de São Paulo Instituto de Medicina Tropical São Paulo SP Brasil Instituto de Medicina Tropical, Universidade de São Paulo, São Paulo, SP – Brasil; 3 Universidade de São Paulo Instituto do Coração do Hospital das Clínicas Departamento Cardiopulmonar São Paulo SP Brasil Departamento Cardiopulmonar, Instituto do Coração do Hospital das Clínicas da Faculdade de Medicina da Universidade de São Paulo, São Paulo, SP – Brasil

**Keywords:** Menopausa, Saúde Bucal, Saliva, Glândulas Salivares, Saúde Vascular

## Abstract

**Fundamento:**

A menopausa representa uma importante transição biológica na vida da mulher, definida pela cessação da função ovariana e por uma redução acentuada dos níveis de estrogênio. Embora suas consequências sistêmicas, incluindo osteoporose e aumento do risco cardiovascular, estejam bem estabelecidas, seu impacto na saúde oral permanece insuficientemente reconhecido tanto por profissionais médicos quanto odontológicos.

**Objetivo:**

Examinar a influência da menopausa na saúde oral das mulheres, enfatizando os principais mecanismos fisiopatológicos, as manifestações clínicas e as estratégias interdisciplinares de prevenção e manejo.

**Métodos:**

Foi realizada uma revisão narrativa utilizando artigos recuperados da base de dados PubMed, publicados entre 2000 e 2025. Foram utilizados os seguintes descritores: "menopause", "oral health", "hormonal changes", "cardiovascular disease", "xerostomia", "salivary glands", "periodontitis" e "postmenopausal women".

**Resultados:**

As alterações hormonais durante a menopausa estão associadas a diversas manifestações orais, incluindo xerostomia, síndrome da ardência bucal, doença periodontal e alterações do paladar. A deficiência de estrogênio parece contribuir para a redução do fluxo salivar, atrofia da mucosa e aumento da resposta inflamatória nos tecidos periodontais. Evidências emergentes também sugerem uma associação entre a saúde oral na menopausa e a doença cardiovascular, entre outras condições sistêmicas. No entanto, persistem lacunas importantes no conhecimento quanto ao manejo clínico e ao cuidado interdisciplinar de mulheres na menopausa.

**Conclusão:**

A saúde oral durante a menopausa reflete uma interação complexa de fatores hormonais, sistêmicos e comportamentais. Diante do aumento da expectativa de vida em todo o mundo, programas de educação e pesquisa em saúde oral devem priorizar esse tema. O fortalecimento da conscientização e a promoção da colaboração multidisciplinar são essenciais para melhorar os desfechos em saúde de mulheres na meia-idade.

## Introdução

A menopausa representa um importante marco biológico na vida da mulher. É caracterizada por um declínio progressivo dos níveis circulantes de estrogênio e progesterona, culminando na cessação permanente da menstruação por pelo menos 12 meses consecutivos. Esse processo resulta da interrupção da função folicular ovariana.^1-3^ A transição geralmente ocorre entre 45 e 55 anos de idade e é acompanhada por uma série de alterações sistêmicas impulsionadas por mudanças nos níveis dos hormônios sexuais femininos. De acordo com o *Red Latinoamericana de Investigación en Climaterio*, a idade média da menopausa na região é de 48,6 anos.^4^

A transição para a menopausa, conhecida como período climatérico, compreende três fases principais. A perimenopausa refere-se aos anos que antecedem a menopausa, durante os quais começam as irregularidades menstruais e frequentemente surgem sintomas vasomotores, como fogachos e sudorese noturna. A menopausa é definida retrospectivamente após 12 meses consecutivos sem menstruação. A pós-menopausa abrange os anos subsequentes à menopausa, período em que os níveis de estrogênio permanecem persistentemente baixos. Apesar desses marcos clínicos, as fases iniciais ou prematuras do climatério ainda não estão completamente definidas.^3-6^

A menopausa afeta significativamente a qualidade de vida e está associada à alteração da homeostase sistêmica, distúrbios do sono, disfunção sexual, ansiedade, depressão, dor musculoesquelética, redução da massa muscular e leve declínio cognitivo.^6,7^ Embora as manifestações somáticas e psicológicas da menopausa, como sintomas vasomotores, distúrbios do sono e alterações de humor, estejam bem documentadas, seu impacto na saúde oral permanece sub-reconhecido tanto na prática clínica quanto nas políticas de saúde pública.

O estrogênio desempenha papel central na manutenção da integridade estrutural e funcional de múltiplos sistemas, incluindo os sistemas cardiovascular, esquelético, tegumentar e os tecidos orais. O estrogênio plasmático circulante existe em três formas principais: 17β-estradiol (E2), estrona e estriol. O E2 é a forma mais potente e biologicamente ativa, sendo predominantemente produzido pelos ovários em mulheres na pré-menopausa. Em mulheres na pós-menopausa, a estrona, forma menos potente, torna-se o principal estrogênio circulante. Os níveis de ambos os hormônios diminuem aproximadamente dez vezes após a menopausa. Durante esse período, a aromatase expressa no tecido adiposo, na pele, no cérebro e no osso torna-se a principal fonte de E2 e estrona circulantes.^8^

A deficiência de estrogênio contribui para um estado de inflamação sistêmica crônica de baixo grau e está associada a condições como doença cardiovascular, osteoporose e atrofia urogenital. Também compromete a homeostase oral.^9^ Nas últimas décadas, o interesse científico pelas consequências orais da menopausa tem crescido, especialmente no contexto do envelhecimento populacional global. A menopausa pode desencadear ou agravar diversas condições orais, incluindo xerostomia, síndrome da ardência bucal (SAB), periodontite, atrofia da mucosa e perda óssea alveolar. Essas alterações podem prejudicar funções orais essenciais, como alimentação, fala e deglutição, além de influenciar a saúde sistêmica.^1,2,10-12^

A relação entre menopausa e saúde oral vai além de manifestações localizadas. Evidências emergentes destacam uma associação bidirecional entre saúde oral e saúde sistêmica, particularmente com condições cardiovasculares, que são mais prevalentes em mulheres na pós-menopausa.^13-16^ Assim, ampliar o conhecimento sobre as manifestações orais da menopausa e integrar o cuidado odontológico às estratégias abrangentes de saúde da mulher constituem prioridades importantes.

Esta revisão narrativa, baseada em artigos recuperados do PubMed publicados entre 2000 e 2025, utilizando os descritores "menopause", "oral health", "hormonal changes", "cardiovascular disease", "xerostomia", "salivary glands", "periodontitis" e "postmenopausal women", tem como objetivo sintetizar as evidências atuais sobre os mecanismos biológicos, as manifestações clínicas e as perspectivas ampliadas da saúde oral em mulheres na menopausa. Também enfatiza a necessidade de cuidado interdisciplinar e de direções futuras de pesquisa para aprimorar a prevenção, o diagnóstico e o manejo (Figura Central).

**Figure:**
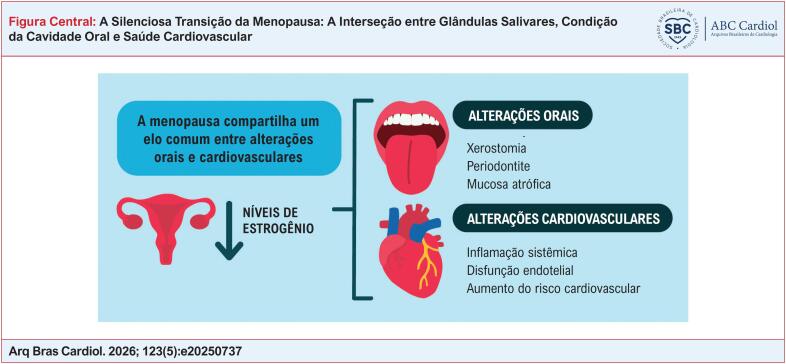


### Menopausa, saliva e glândulas salivares: associações com alterações orais comuns

A cavidade oral não é tradicionalmente considerada um alvo primário dos hormônios sexuais; no entanto, é altamente sensível às flutuações hormonais.^1,2,9,12,14,17^ Os estrogênios desempenham papel fundamental na manutenção de sua integridade estrutural e funcional. Em condições fisiológicas, tecidos moles, osso alveolar, vasculatura, glândulas salivares e microbioma oral interagem dinamicamente para preservar a homeostase. Durante a menopausa, o declínio progressivo do estrogênio e da progesterona circulantes desencadeia uma cascata de alterações nos tecidos orais, nas glândulas salivares e na regulação imunológica, aumentando coletivamente a suscetibilidade a doenças orais.^10,11,13,14^

Receptores de estrogênio (ERα e ERβ) foram identificados na mucosa oral, nas glândulas salivares e nos tecidos periodontais.^2,12^ Esses receptores mediam a modulação hormonal da proliferação celular, da diferenciação e das respostas inflamatórias. O estrogênio também regula a permeabilidade vascular e o metabolismo do colágeno, processos essenciais para manter a resiliência dos tecidos moles e a integridade do tecido conjuntivo gengival.^18^

Os estrogênios influenciam a função das glândulas salivares ao promover a atividade das células acinares e vascularização. As glândulas parótidas e submandibulares, em particular, parecem responder à modulação hormonal.^19^ Em mulheres na menopausa, níveis reduzidos de estrogênio têm sido associados a alterações relatadas na função das glândulas salivares e na quantidade e qualidade da saliva, embora os achados ainda sejam inconsistentes.^20^

A saliva é um ultrafiltrado plasmático processado pelas glândulas salivares e é essencial para a manutenção e proteção das estruturas orais. Desempenha papel central na homeostase oral. A saliva humana contém numerosos componentes conhecidos e ainda não identificados que contribuem para a capacidade tampão, lubrificação, defesa antimicrobiana e proteção tecidual. Entre eles estão mieloperoxidase, óxido nítrico (NO), imunoglobulina A (IgA), enzimas e eletrólitos.^21,22^

O NO é um potente vasodilatador e desempenha papel na regulação da função dos cardiomiócitos, entre outros efeitos sistêmicos. Sua concentração na saliva excede a encontrada no plasma. O NO também atua como molécula sinalizadora, neurotransmissor e mediador na defesa imune e nos processos inflamatórios.^23^ Contribui para a secreção salivar e é considerado molécula-chave na manutenção da homeostase. O nitrato da dieta (exógeno) e o nitrato derivado da oxidação endógena do NO são absorvidos pelo trato gastrointestinal para a corrente sanguínea. Aproximadamente 25% do nitrato circulante é captado ativamente pelas glândulas salivares, especialmente pela glândula parótida, e secretado na saliva. Desse total, cerca de 20% é reduzido a nitrito por bactérias orais. O nitrato e o nitrito restantes são deglutidos e retornam à circulação sistêmica, compondo o ciclo enterossalivar nitrato-nitrito-NO. Notavelmente, aproximadamente 80% do nitrito circulante é gerado por essa via.^23^ As concentrações salivares de nitrato são cerca de dez vezes superiores às plasmáticas.^24^

Abadi et al. demonstraram que os níveis salivares de NO são significativamente menores em pacientes diabéticos com xerostomia em comparação com aqueles sem xerostomia, sugerindo que o NO pode contribuir para a manutenção da saúde oral.^25^ No entanto, a relação entre o metabolismo do NO, as alterações das glândulas salivares durante a menopausa e a função cardiovascular permanece insuficientemente explorada.

Apesar de a xerostomia ser uma das queixas mais frequentemente relatadas por mulheres na menopausa, os estudos que avaliam a taxa de fluxo salivar e a resposta das glândulas salivares nesse período ainda são limitados.^26^ Xerostomia é definida como a sensação subjetiva de boca seca e não necessariamente se correlaciona com reduções mensuráveis do fluxo salivar. Embora multifatorial, acredita-se que o hipoestrogenismo prejudique a função das glândulas salivares, potencialmente reduzindo tanto o fluxo salivar não estimulado quanto o estimulado.

Em um estudo post mortem, Moraes et al. observaram alterações vasculares nas glândulas salivares de mulheres de meia-idade e idosas, incluindo trombose com comprometimento acentuado da perfusão.^27^ Essas alterações vasculares podem prejudicar a nutrição do parênquima, levando à atrofia glandular e à substituição por tecido adiposo. Kawamoto et al. corroboraram esses achados, relatando alterações na composição do parênquima salivar e substituição adiposa associadas à hipertensão, uma comorbidade comum em mulheres na menopausa.^16,27^

Clinicamente, a xerostomia em mulheres na menopausa está associada a prejuízo da fala, mastigação e deglutição. A redução do fluxo salivar e alterações na composição podem resultar em pH salivar mais baixo, menor capacidade tampão e atividade antimicrobiana diminuída.^20^ Em conjunto, essas alterações criam um ambiente mais suscetível à cárie dentária, infecções orais e halitose.^2,28,29^ A xerostomia também pode afetar negativamente as interações sociais, e sua gravidade parece correlacionar-se com a duração da menopausa.^10^

As pesquisas permanecem inconsistentes. Alguns autores relatam redução do fluxo salivar após a menopausa, enquanto outros descrevem alterações na composição salivar sem redução mensurável do fluxo.^20,22,30^ Quando presente, a redução do fluxo salivar não estimulado e estimulado contribui para xerostomia e aumenta o risco de cárie dentária, candidose e desconforto mucoso.^1^ Além disso, reduções na capacidade tampão e em componentes antimicrobianos, como lisozima, lactoferrina e IgA, podem comprometer ainda mais os mecanismos de defesa oral.^20^

Salvolini et al. não observaram diferenças significativas na produção salivar em pacientes idosas, mas relataram níveis aumentados de peróxidos lipídicos, sugerindo maior estresse oxidativo e possível dano tecidual.^22^ Além disso, a saliva de mulheres na pós-menopausa tem sido associada a valores de pH mais baixos correlacionados com níveis plasmáticos de adiponectina, marcador ligado ao risco cardiometabólico pró-inflamatório e a distúrbios metabólicos como diabetes tipo 2 e obesidade.^21,31^

A xerostomia predispõe a alterações mucosas. A combinação de redução do fluxo salivar, atrofia da mucosa e alterações das respostas imunes favorece infecções oportunistas, particularmente candidose oral.^1^ Embora geralmente manejável, a candidose recorrente pode servir como indicador clínico de xerostomia subjacente ou desequilíbrio sistêmico em mulheres na menopausa.^2,8,27,28^

Semelhante à mucosa genital, a mucosa oral pode tornar-se mais fina, menos elástica e mais suscetível a traumas após o declínio do estrogênio. As pacientes podem relatar sensação de repuxamento, desconforto ao consumir alimentos ácidos ou condimentados, ou maior sensibilidade a variações de temperatura. Achados clínicos podem incluir glossite atrófica (língua lisa e despapilada), queilite angular e palidez da mucosa. Essas alterações podem ser exacerbadas pela redução da lubrificação salivar.^1,2^

A menopausa também pode estar associada a distúrbios sensoriais, incluindo SAB e disgeusia. A alteração da percepção do paladar pode estar relacionada a mudanças na taxa de fluxo salivar e no processamento central dos estímulos gustativos. As alterações do paladar são frequentemente subnotificadas; algumas mulheres descrevem sensações metálicas ou amargas persistentes, enquanto outras relatam redução da sensibilidade aos sabores doce ou salgado. Os mecanismos propostos incluem alterações na composição da saliva, mudanças na densidade dos receptores gustativos e efeitos hormonais sobre as vias gustativas centrais.^2^

A SAB é definida como uma condição de dor neuropática crônica caracterizada por sensação de queimação na ausência de lesões mucosas visíveis ou alterações laboratoriais. Foi relatada em até 18% das mulheres na pós-menopausa e comumente afeta língua, lábios e palato duro. A etiologia permanece incompletamente compreendida, mas acredita-se envolver disfunção neuropática periférica ou central modulada por fatores hormonais, psicológicos e sistêmicos.^1^ Condições psiquiátricas e polimorfismos genéticos podem contribuir. Fatores sistêmicos associados à SAB incluem diabetes mellitus, deficiências de vitaminas do complexo B (B1, B2, B6 e B12), folato e ferro, distúrbios gastrointestinais, desequilíbrios hormonais e efeitos relacionados a medicamentos. A maior prevalência de SAB em mulheres na menopausa levou à hipótese de que estrogênio e progesterona possam exercer efeitos neuroprotetores ou moduladores neurossensoriais que diminuem após a menopausa. Os sintomas geralmente surgem entre 3 e 12 anos após o início da menopausa.^17^

Os estudos existentes são limitados por tamanhos amostrais reduzidos e heterogeneidade metodológica. São necessárias pesquisas adicionais para elucidar os mecanismos subjacentes à senescência das glândulas salivares e às alterações funcionais durante a menopausa. Além disso, comorbidades relacionadas à idade e medicamentos, como anti-hipertensivos, antidepressivos e anticolinérgicos, podem influenciar adicionalmente o fluxo salivar nessa população.

Um resumo integrado dos achados salivares e periodontais ao longo dos estágios da menopausa é apresentado na Tabela 1.

**Tabela 1:** Panorama de estudos clínicos e experimentais sobre alterações relacionadas à menopausa na função das glândulas salivares, homeostase oral e saúde periodontal

ID do estudo	População (n, status)	Metodologia	Principais achados	Relevância translacional
Tremblay et al., 2012^31^	151 mulheres (53 pré-menopausa; 98 pós-menopausa)	pH da STNE; adiponectina plasmática (ELISA); medidas antropométricas	pH salivar correlacionou-se positivamente com adiponectina plasmática em mulheres na menopausa	Níveis mais baixos de adiponectina e pH salivar reduzido foram observados em mulheres na menopausa, sugerindo associação com perfil cardiometabólico pró-inflamatório; requer validação longitudinal
Salvolini et al., 2000^22^	169 indivíduos saudáveis de ambos os sexos divididos em cinco faixas etárias (10-24 anos, n = 38; 25-39 anos, n = 52; 40-54 anos, n = 40; 55-69 anos, n = 27; ≥ 70 anos, n = 12)	Avaliação de proteína total, atividade da peroxidase, malondialdeído e níveis de NO na STNE	Sem diferenças no volume salivar; redução da atividade da peroxidase e aumento dos níveis de NO com o envelhecimento	Sugere estresse oxidativo relacionado à idade na saliva independentemente do volume salivar; ausência de perfil hormonal direto
Foglio-Bonda et al., 2019^37^	120 mulheres (60 pós-menopausa; 60 férteis)	pH e taxa de fluxo da STNE; CPOD	pH salivar mais baixo e fluxo reduzido no grupo menopausa; índice CPOD semelhante	Coleta padronizada de STNE demonstra hipofunção salivar modesta, porém significativa
Bosman et al., 2022^43^	50 mulheres saudáveis (24 com 22-45 anos; 26 com 55-92 anos)	STNE; metabolômica não direcionada via CL-EM	Alterações no perfil metabolômico salivar associadas à idade	Metabólitos diferencialmente expressos em mulheres mais velhas relacionados ao metabolismo de aminoácidos, ciclo de Krebs, metabolismo de ácidos graxos e síntese de ácidos nucleicos
Soundarya et al., 2022^36^	60 mulheres (20 menstruadas, 20 pré-menopausa, 20 pós-menopausa)	STNE; estradiol salivar (ELISA); cálcio salivar; índices de xerostomia e periodontais	Menores níveis de estradiol salivar e maiores níveis de cálcio no grupo pós-menopausa; estradiol reduzido associado a maiores escores de xerostomia e doença periodontal	Potenciais marcadores de triagem; requer padronização
Harika et al., 2024^26^	60 mulheres (30 pré-menopausa; 30 pós-menopausa)	STNE; limiares gustativos; níveis séricos de zinco	Mulheres pós-menopausa apresentaram redução na percepção dos sabores doce e amargo; sem diferenças no fluxo salivar ou níveis de zinco	Alterações do paladar podem ser multifatoriais, além da deficiência de zinco ou redução do fluxo; considerar fatores neurossensoriais
Moraes et al., 2024^27^	85 mulheres submetidas à autópsia (fase reprodutiva ≤ 44 anos, n = 18; climatério 45-55 anos, n = 21; pós-menopausa ≥ 56 anos, n = 46)	Análise morfológica de glândulas salivares menores labiais	Envelhecimento associado a fibrose e atrofia acinar, metaplasia serosa, substituição adiposa e microtrombose	Fornece base anatômica para hipofunção salivar; sem correlação com sintomas clínicos
García-Alfaro et al., 2025^42^	3.211 mulheres peri- (n = 1.170) e pós-menopausa (n = 2.041), 40-90 anos	IX; qualidade de vida relacionada à saúde oral (OHIP-14)	Maior prevalência de xerostomia em mulheres peri- e pós-menopausa; associada a pior qualidade de vida	Apoia triagem rotineira de xerostomia em serviços focados na menopausa
Scardina & Messina, 2012^44^	27 mulheres pré-menopausa e 27 pós-menopausa	Avaliação da microcirculação oral (videocapilaroscopia); índices periodontais	Redução da densidade capilar periodontal, aumento da tortuosidade vascular e diminuição do diâmetro das alças em mulheres pós-menopausa	Sugere contribuição vascular para maior vulnerabilidade periodontal
LaMonte et al., 2013^45^	1.025 mulheres pós-menopausa (acompanhamento de 5 anos)	Avaliação longitudinal da progressão da doença periodontal	Progressão associada a fatores sistêmicos; ausência de progressão generalizada; mulheres com periodontite grave prévia apresentaram perda óssea acelerada	Estudo de coorte robusto; indica necessidade de estratificação de risco
Wactawski-Wende et al., 2020^14^	1.342 mulheres pós-menopausa na linha de base; 1.026 (acompanhamento de 5 anos); 518 (acompanhamento de 5 anos)	Exames orais abrangentes; densidade óssea e composição corporal; microbioma subgengival (sequenciamento 16S)	Achados integrados orais e sistêmicos em mulheres envelhecendo	Base de dados de referência para modelos de risco oral-sistêmico
Ahmad et al., 2024^39^	100 mulheres pós-menopausa	Exame periodontal completo; densitometria óssea	Idade identificada como fator de risco significativo para perda óssea em mulheres pós-menopausa	Apoia triagem periodontal baseada em risco; desenho observacional limita inferência causal
Yakar et al., 2024^46^	75 pós-menopausa (38-65 anos); 71 pré-menopausa (33-52 anos)	Citocinas no soro e saliva (TNF-α, IL-6, IL-10, IL-7); IMC; *Women's Health Questionnaire*; avaliação periodontal	Sem diferenças significativas nas citocinas após ajuste por idade; menos dentes no grupo pós-menopausa; sem diferenças nos parâmetros periodontais	Idade como importante fator de confusão; citocinas salivares isoladamente podem não diferenciar *status* menopausal; reforça etiologia multifatorial
Yakar et al., 2025^47^	77 pré-menopausa; 81 pós-menopausa	Hibridização DNA-DNA em painel (40 espécies) e sequenciamento 16S; estradiol sérico (ELISA); SMDI modificado	Apesar de periodontite mais grave, não foi observada disbiose microbiana relacionada à menopausa; duas espécies correlacionaram-se com estradiol na pré-menopausa	Variação microbiana parece associada à gravidade da doença e não ao status hormonal; destaca regulação sistêmica da ecologia oral

16S: sequenciamento do gene do RNA ribossômico 16S; CL-EM: cromatografia líquida acoplada à espectrometria de massas; CPOD: índice de Dentes Cariados, Perdidos e Obturados; ELISA: ensaio imunoenzimático; IL-6: interleucina 6; IL-7: interleucina 7; IL-10: interleucina 10; IMC: índice de massa corporal; IX: Inventário de Xerostomia; NO: óxido nítrico; OHIP-14: Oral Health Impact Profile, versão de 14 itens; SMDI: Subgingival Microbial Dysbiosis Index; STNE: saliva total não estimulada; TNF-α: fator de necrose tumoral alfa.

### Saúde oral, menopausa e doença cardiovascular

O declínio do estrogênio durante a menopausa afeta não apenas a cavidade oral, mas também acelera o risco cardiovascular, que permanece como a principal causa de morte em mulheres. Mulheres em idade reprodutiva apresentam menor incidência de doença cardiovascular em comparação aos homens; no entanto, essa diferença diminui após a menopausa, quando o risco cardiovascular aumenta substancialmente.^8,32^ A transição menopausal representa, portanto, uma etapa crucial na saúde cardiometabólica feminina.

O hipoestrogenismo na menopausa está associado à redução da biodisponibilidade de NO, molécula com propriedades anti-inflamatórias, antioxidantes e antiaterogênicas.^18^ Ao mesmo tempo, a menopausa é caracterizada por aumento na produção de mediadores pró-inflamatórios, incluindo fator de necrose tumoral-α, interleucina-6 e proteína C reativa. Essas citocinas, inclusive aquelas originadas de tecidos periodontais inflamados, podem alcançar a circulação sistêmica e contribuir para disfunção endotelial, aterogênese e instabilidade de placas, mecanismos centrais na patologia cardiovascular.

No sistema cardiovascular, a menopausa está associada à disfunção endotelial e da musculatura lisa vascular, além de aterosclerose microvascular. Essas alterações podem levar à doença arterial coronariana não obstrutiva mediada por disfunção microvascular coronariana.^17^ Nos Estados Unidos, mulheres na pós-menopausa com deficiência de estrogênio representam parcela substancial dos pacientes diagnosticados com doença arterial coronariana não obstrutiva.^33^

Em paralelo às evidências de disfunção microvascular no sistema cardiovascular, Uhl et al. relataram que diversas condições patológicas das glândulas salivares estão associadas à doença microvascular relacionada à inflamação.^34^ No entanto, o impacto inflamatório da menopausa sobre a microvasculatura das glândulas salivares e suas consequências para a composição e o fluxo salivar permanecem insuficientemente investigados. Alguns estudos sugerem que alterações trombóticas microvasculares nas glândulas salivares, combinadas aos efeitos sistêmicos do hipoestrogenismo, como hipertensão, podem comprometer o parênquima glandular. Esse processo pode levar à atrofia glandular e à manifestação clínica de xerostomia.^16,27^

Um corpo crescente de evidências sustenta uma relação interdependente entre menopausa, doença periodontal e risco cardiovascular. O estrogênio exerce efeitos vasodilatadores, anti-inflamatórios e antioxidantes sobre o endotélio vascular, em parte por meio da manutenção dos níveis de NO.^8^ Sua redução durante a menopausa tem sido associada ao aumento da rigidez arterial, alterações adversas no perfil lipídico e mudanças nos padrões de coagulação, fatores que contribuem para maior risco cardiovascular.^35^

Simultaneamente, o hipoestrogenismo promove atrofia da mucosa oral, disfunção salivar e reabsorção óssea alveolar, criando condições favoráveis ao desenvolvimento de doença periodontal.^7,14,36^ Esses mecanismos paralelos sugerem que o estrogênio exerce papel regulador central tanto na saúde oral quanto na saúde cardiovascular. Além disso, alterações no pH salivar têm sido associadas ao estado inflamatório, ao estresse oxidativo e ao metabolismo do NO, com possíveis implicações cardiovasculares sistêmicas.^22,37^

Mulheres na menopausa apresentam maior prevalência e gravidade de gengivite e periodontite. Essas alterações podem resultar de efeitos hormonais diretos, bem como de modificações sistêmicas, incluindo redução da densidade mineral óssea e desregulação da resposta imune.^14,38^ A deficiência de estrogênio parece amplificar a resposta inflamatória aos patógenos periodontais, aumentar a degradação do tecido conjuntivo e comprometer a remodelação do osso alveolar. Esses processos aceleram a perda de inserção periodontal e podem, em última instância, levar à mobilidade e perda dentária quando não tratados.^14,39^

A osteoporose pós-menopausa pode agravar ainda mais a perda óssea alveolar, reforçando um possível elo entre saúde esquelética, inflamação crônica e doença cardiovascular.^1,39,40^ A inflamação crônica de baixo grau associada à periodontite tem sido implicada no desenvolvimento e na progressão da doença arterial coronariana, do acidente vascular cerebral e da doença arterial periférica. Esse processo é iniciado pela disbiose microbiana oral e perpetuado pela resposta imune do hospedeiro.^1,39,41^

Para os clínicos, o reconhecimento dessa tríade, menopausa, doença oral e risco cardiovascular, deve orientar a anamnese e a estratificação de risco de forma abrangente. O estado periodontal pode atuar como marcador de inflamação sistêmica e vulnerabilidade cardiovascular. Por outro lado, sintomas orais inexplicados em mulheres na pós-menopausa podem justificar avaliação metabólica e vascular mais ampla.^40^ Estratégias de saúde pública direcionadas a mulheres de meia-idade devem integrar a promoção da saúde oral aos programas de prevenção cardiovascular, especialmente em populações envelhecidas e socialmente vulneráveis.

Além dos mecanismos inflamatórios, as condições orais e cardiovasculares relacionadas à menopausa compartilham diversos fatores de risco modificáveis, incluindo tabagismo, diabetes mellitus, obesidade, sedentarismo e padrões alimentares não saudáveis. Estresse psicossocial e distúrbios do sono, ambos comuns durante a menopausa, podem intensificar a inflamação sistêmica e reduzir a busca por cuidados em saúde, ampliando o risco tanto de doença periodontal quanto de doença cardiovascular.^1,14,42^

### Lacunas de conhecimento e perspectivas futuras

Apesar do reconhecimento crescente da relação entre menopausa e saúde oral, persistem lacunas substanciais tanto na evidência científica quanto na implementação clínica. Essas limitações dificultam o desenvolvimento de fluxos assistenciais padronizados e de intervenções baseadas em evidências direcionadas às mulheres na menopausa.

A menopausa é uma transição biopsicossocial que vai além das alterações fisiológicas, frequentemente afetando o humor, a cognição e a resiliência emocional. As manifestações orais nesse período podem influenciar significativamente a autoimagem, a participação social e a qualidade de vida. Também podem contribuir para estresse psicológico, ansiedade e sintomas depressivos. Apesar de seu impacto, essas queixas orais são frequentemente subnotificadas pelas pacientes e sub-reconhecidas tanto na prática médica quanto odontológica.

A integridade oral é essencial para mastigação, deglutição, fonação e comunicação não verbal. Sintomas orais da menopausa, especialmente aqueles relacionados à atrofia mucosa, comprometimento dentário ou hipofunção salivar, podem prejudicar essas funções fundamentais. Dificuldades para comer e falar em contextos sociais podem levar ao constrangimento e ao isolamento social. A insatisfação com a estética oral, incluindo escurecimento dentário, perda dentária e alterações da mucosa, pode afetar negativamente a autoestima e a imagem corporal, que já podem estar fragilizadas nessa fase da vida.

O enfrentamento dessas lacunas exige uma agenda de pesquisa multidisciplinar coordenada, políticas públicas de apoio e a transição para modelos assistenciais centrados na paciente e baseados no curso de vida. A separação histórica entre odontologia e medicina permanece como importante barreira para o manejo integral. Embora sintomas orais sejam comuns na menopausa, poucas mulheres recebem avaliação odontológica estruturada como parte do acompanhamento ginecológico, cardiológico ou da atenção primária. Da mesma forma, profissionais da odontologia podem não dispor de protocolos padronizados para rastrear condições sistêmicas, como osteoporose, doença cardiovascular ou síndrome metabólica, que influenciam a saúde oral.

Tecnologias emergentes, incluindo diagnósticos salivares, sequenciamento do microbioma e avaliação periodontal digital, oferecem ferramentas promissoras para identificação mais precoce e precisa das condições orais relacionadas à menopausa. São necessárias pesquisas adicionais para validar essas abordagens em populações menopáusicas diversas e para avaliar sua aplicabilidade clínica na prática rotineira.

Uma melhor compreensão da relação entre distúrbios orais e sintomas cardiovasculares, como angina ou alterações isquêmicas cardíacas, também pode contribuir para uma estratificação mais abrangente do risco cardiovascular em mulheres na menopausa.

## Data Availability

Os conteúdos subjacentes ao texto da pesquisa estão contidos no manuscrito.
