# Supplementary material for: A Silenciosa Transição da Menopausa: A Interseção entre Glândulas Salivares, Condição da Cavidade Oral e Saúde Cardiovascular
Source: Arq Bras Cardiol. 2026 May 18;123(5):e20250737. [Article in Portuguese] doi: 10.36660/abc.20250737 (PMC13399042; doi:10.36660/abc.20250737)
Supplement: Translation

# Translation: The Silent Shift of Menopause: The Intersection between Salivary Glands, Oral Cavity Status, and Cardiovascular Health

**DOI:** 10.36660/abc.20250737i

**Authors:** Silvia Vanessa Lourenço, Giovanna Piacenza Florezi, Cibele Pelissari, Flavio Carneiro Hojaij, José Pinhata Otoch, José Antonio Franchini Ramires

**Affiliations:** 1 São Paulo SP Brazil Departamento de Cirurgia, Faculdade de Medicina, Universidade de São Paulo, São Paulo, SP – Brazil; 2 São Paulo SP Brazil Instituto de Medicina Tropical, Universidade de São Paulo, São Paulo, SP – Brazil; 3 São Paulo SP Brazil Departamento Cardiopulmonar, Instituto do Coração do Hospital das Clínicas da Faculdade de Medicina da Universidade de São Paulo, São Paulo, SP – Brazil

**Keywords:** Menopause, Oral Health, Saliva, Salivary Glands, Vascular Health

## Abstract

**Background:**

Menopause represents a major biological transition in a woman's life, defined by the cessation of ovarian function and a marked decline in estrogen levels. Although its systemic consequences, including osteoporosis and increased cardiovascular risk, are well established, its impact on oral health remains insufficiently recognized by both medical and dental professionals.

**Objective:**

To examine the influence of menopause on women's oral health, emphasizing key pathophysiological mechanisms, clinical manifestations, and interdisciplinary strategies for prevention and management.

**Methods:**

A narrative review was conducted using articles retrieved from the PubMed database published between 2000 and 2025. The following keywords were used: "menopause," "oral health," "hormonal changes," "cardiovascular disease," "xerostomia," "salivary glands," "periodontitis," and "postmenopausal women."

**Results:**

Hormonal changes during menopause are associated with a range of oral manifestations, including xerostomia, burning mouth syndrome, periodontal disease, and taste alterations. Estrogen deficiency appears to contribute to reduced salivary flow, mucosal atrophy, and an increased inflammatory response in periodontal tissues. Emerging evidence also suggests an association between oral health during menopause and cardiovascular disease, among other systemic conditions. However, important knowledge gaps persist regarding clinical management and interdisciplinary care for menopausal women.

**Conclusion:**

Oral health during menopause reflects a complex interplay of hormonal, systemic, and behavioral factors. Because of increasing life expectancy worldwide, oral health education and research programs should prioritize this topic. Strengthening awareness and fostering multidisciplinary collaboration are essential to improving health outcomes in midlife women.

## Introduction

Menopause represents a major biological milestone in a woman's life. It is characterized by a progressive decline in circulating estrogen and progesterone levels, culminating in the permanent cessation of menstruation for at least 12 consecutive months. This process results from the cessation of ovarian follicular function.^1-3^ The transition typically occurs between 45 and 55 years of age and is accompanied by a range of systemic changes driven by alterations in female sex hormone levels. According to the Collaborative Group for Research of the Climacteric in Latin America, the average age of menopause in the region is 48.6 years.^4^

The transition into menopause, known as the climacteric period, comprises three principal phases. Perimenopause refers to the years preceding menopause, during which menstrual irregularities begin and are often accompanied by vasomotor symptoms such as hot flashes and night sweats. Menopause is defined retrospectively after 12 consecutive months without menstruation. Postmenopause encompasses the years following menopause, during which estrogen levels remain persistently low. Despite these clinical landmarks, the early or premature phases of the climacteric period remain incompletely defined.^3-6^

Menopause significantly affects quality of life and is associated with altered systemic homeostasis, sleep disturbances, sexual dysfunction, anxiety, depression, musculoskeletal pain, reduced muscle mass, and mild cognitive decline.^6,7^ Although the somatic and psychological manifestations of menopause, such as vasomotor symptoms, sleep disturbances, and mood changes, are well documented, its impact on oral health remains underrecognized in both clinical practice and public health settings.

Estrogen plays a central role in maintaining the structural and functional integrity of multiple systems, including the cardiovascular, skeletal, integumentary, and oral tissues. Circulating plasma estrogen exists in three main forms: 17β-estradiol (E2), estrone, and estriol. E2 is the most potent and biologically active form and is predominantly produced by the ovaries in premenopausal women. In postmenopausal women, estrone, a less potent form, becomes the predominant circulating estrogen. The levels of both hormones decrease approximately tenfold after menopause. During this period, aromatase expressed in adipose tissue, skin, brain, and bone becomes the primary source of circulating E2 and estrone.^8^

Estrogen deficiency contributes to a state of low-grade chronic systemic inflammation and is associated with conditions such as cardiovascular disease, osteoporosis, and urogenital atrophy. It also disrupts oral homeostasis.^9^ In recent decades, growing scientific interest has focused on the oral consequences of menopause, particularly in the context of global population aging. Menopause may precipitate or exacerbate several oral conditions, including xerostomia, burning mouth syndrome (BMS), periodontitis, mucosal atrophy, and loss of alveolar bone. These alterations may impair essential oral functions such as eating, speaking, and swallowing and may also influence systemic health.^1,2,10-12^

The relationship between menopause and oral health extends beyond localized manifestations. Emerging evidence highlights a bidirectional association between oral and systemic health, particularly with cardiovascular conditions, which are more prevalent in postmenopausal women.^13-16^ Thus, improving knowledge of the oral manifestations of menopause and integrating oral care into comprehensive women's health strategies represent important priorities.

This narrative review, based on articles retrieved from PubMed published between 2000 and 2025 using the keywords "menopause," "oral health," "hormonal changes," "cardiovascular disease," "xerostomia," "salivary glands," "periodontitis," and "postmenopausal women," aims to synthesize current evidence on the biological mechanisms, clinical manifestations, and broader perspectives of oral health in menopausal women. It also emphasizes the need for interdisciplinary care and future research directions to improve prevention, diagnosis, and management (Central Illustration).

### Menopause, saliva, and salivary glands: associations with common oral changes

The oral cavity is not traditionally considered a primary target of sex hormones; however, it is highly sensitive to hormonal fluctuations.^1,2,9,12,14,17^ Estrogens play a fundamental role in maintaining its structural and functional integrity. Under physiological conditions, soft tissues, alveolar bone, vasculature, salivary glands, and the oral microbiome interact dynamically to preserve homeostasis. During menopause, the progressive decline in circulating estrogen and progesterone triggers a cascade of changes in oral tissues, salivary glands, and immune regulation, collectively increasing susceptibility to oral diseases.^10,11,13,14^

Estrogen receptors (ERα and ERβ) have been identified in the oral mucosa, salivary glands, and periodontal tissues.^2,12^ These receptors mediate hormonal modulation of cellular proliferation, differentiation, and inflammatory responses. Estrogen also regulates vascular permeability and collagen metabolism, processes that are essential for maintaining soft tissue resilience and gingival connective tissue integrity.^18^

Estrogens influence salivary gland function by promoting acinar cell activity and vascularization. The parotid and submandibular glands, in particular, appear responsive to hormonal modulation.^19^ In menopausal women, reduced estrogen levels have been associated with reported alterations in salivary gland function and in both the quantity and quality of saliva, although findings remain inconsistent.^20^

Saliva is a plasma ultrafiltrate processed by the salivary glands and is essential for maintaining and protecting oral structures. It plays a central role in oral homeostasis. Human saliva contains numerous known and yet-to-be-identified components that contribute to buffering capacity, lubrication, antimicrobial defense, and tissue protection. These include myeloperoxidase, nitric oxide (NO), immunoglobulin A (IgA), enzymes, and electrolytes.^21,22^

NO is a potent vasodilator and plays a role in regulating cardiomyocyte function, among other systemic effects. Its concentration in saliva exceeds that found in plasma. NO also functions as a signaling molecule, neurotransmitter, and mediator in immune defense and inflammatory processes.^23^ It contributes to salivary secretion and is considered a key molecule in maintaining homeostasis. Dietary nitrate (exogenous) and nitrate derived from endogenous NO oxidation are absorbed through the gastrointestinal tract into the bloodstream. Approximately 25% of circulating nitrate is actively taken up by the salivary glands, particularly the parotid gland, and secreted into saliva. Of this amount, about 20% is reduced to nitrite by oral bacteria. The remaining nitrate and nitrite are swallowed and re-enter systemic circulation, forming part of the enterosalivary nitrate-nitrite-NO cycle. Notably, approximately 80% of circulating nitrite is generated through this pathway.^23^ Salivary nitrate concentrations are approximately 10-fold higher than plasma levels.^24^

Abadi et al. demonstrated that salivary NO levels are significantly lower in diabetic patients with xerostomia compared with those without xerostomia, suggesting that NO may contribute to oral health maintenance.^25^ However, the relationship between NO metabolism, salivary gland alterations during menopause, and cardiovascular function remains insufficiently explored.

Despite xerostomia being one of the most frequently reported complaints among menopausal women, studies evaluating salivary flow rate and salivary gland response during menopause remain limited.^26^ Xerostomia is defined as the subjective sensation of oral dryness and is not necessarily correlated with measurable reductions in salivary flow. Although multifactorial, hypoestrogenism is thought to impair salivary gland function, potentially reducing both unstimulated and stimulated salivary flow.

In a postmortem study, de Moraes et al. observed vascular alterations in the salivary glands of midlife and older women, including thrombosis with marked perfusion compromise.^27^ These vascular changes may impair parenchymal nutrition, leading to glandular atrophy and replacement by adipose tissue. Kawamoto et al. corroborated these findings, reporting alterations in salivary parenchymal composition and adipose replacement associated with hypertension, a common comorbidity in menopausal women.^16,27^

Clinically, xerostomia in menopausal women is associated with impairment of speech, mastication, and swallowing. Reduced salivary output and compositional changes may result in lower salivary pH, decreased buffering capacity, and diminished antimicrobial activity.^20^ Together, these alterations create an environment more susceptible to dental caries, oral infections, and halitosis.^2,28,29^ Xerostomia may also negatively affect social interactions, and its severity appears to correlate with menopause duration.^10^

Research remains inconsistent. Some authors report reduced salivary flow after menopause, whereas others describe changes in salivary composition without measurable flow reduction.^20,22,30^ When present, reduced unstimulated and stimulated salivary flow contributes to xerostomia and increases the risk of dental caries, candidosis, and mucosal discomfort.^1^ Additionally, reductions in buffering capacity and antimicrobial components, such as lysozyme, lactoferrin, and IgA, may further compromise oral defense mechanisms.^20^

Salvolini et al. did not observe significant differences in salivary output in older patients but reported increased lipid peroxide levels, suggesting enhanced oxidative stress and potential tissue damage.^22^ Moreover, postmenopausal saliva has been associated with lower pH values correlated with plasma adiponectin levels, a marker linked to pro-inflammatory cardiometabolic risk and metabolic disorders such as type 2 diabetes and obesity.^21,31^

Xerostomia predisposes to mucosal alterations. The combination of reduced salivary flow, mucosal atrophy, and altered immune responses favors opportunistic infections, particularly oral candidosis.^1^ Although typically manageable, recurrent candidosis may serve as a clinical indicator of underlying xerostomia or systemic imbalance in menopausal women.^2,8,27,28^

Similar to the genital mucosa, the oral mucosa may become thinner, less elastic, and more susceptible to trauma following estrogen decline. Patients may report tightness, discomfort when consuming acidic or spicy foods, or increased sensitivity to temperature changes. Clinical findings may include atrophic glossitis (smooth, depapillated tongue), angular cheilitis, and mucosal pallor. These alterations may be exacerbated by reduced salivary lubrication.^1,2^

Menopause may also be associated with sensory disturbances, including BMS and dysgeusia. Altered taste perception may be related to changes in salivary flow rate and central processing of gustatory stimuli. Taste disturbances are often underreported; some women describe persistent metallic or bitter sensations, whereas others experience reduced sensitivity to sweet or salty tastes. Proposed mechanisms include changes in salivary composition, alterations in taste receptor density, and hormonal effects on central gustatory pathways.^2^

BMS is defined as a chronic neuropathic pain condition characterized by burning sensations in the absence of visible mucosal lesions or laboratory abnormalities. It has been reported in up to 18% of postmenopausal women and commonly affects the tongue, lips, and anterior palate. The etiology remains incompletely understood but is thought to involve peripheral or central neuropathic dysfunction modulated by hormonal, psychological, and systemic factors.^1^ Psychiatric conditions and genetic polymorphisms may contribute. Systemic factors associated with BMS include diabetes mellitus, deficiencies of B vitamins (B1, B2, B6, and B12), folate, and iron, gastrointestinal disorders, hormonal imbalances, and medication-related effects. The higher prevalence of BMS in menopausal women has led to the hypothesis that estrogen and progesterone may exert neuroprotective or neurosensory modulatory effects that diminish after menopause. Symptoms typically emerge 3 to 12 years after menopause onset.^17^

Existing studies are limited by small sample sizes and methodological heterogeneity. Further research is needed to elucidate the mechanisms underlying salivary gland senescence and functional alterations during menopause. In addition, age-related comorbidities and medications, such as antihypertensives, antidepressants, and anticholinergics, may further influence salivary flow in this population.

An integrated summary of salivary and periodontal findings across menopausal stages is presented in Table 1.

**Table 1:** Overview of clinical and experimental studies on menopause-related changes in salivary gland function, oral homeostasis, and periodontal health

Study ID	Population (n, status)	Methodology	Key findings	Translational relevance
Tremblay et al., 2012^31^	151 women (53 premenopausal; 98 postmenopausal)	UWS pH; plasma adiponectin (ELISA); anthropometric measurements	Salivary pH positively correlated with plasma adiponectin in menopausal women	Lower adiponectin levels and reduced salivary pH were observed in menopausal women, suggesting an association with a proinflammatory cardiometabolic risk profile; requires longitudinal validation
Salvolini et al., 2000^22^	169 healthy subjects of both sexes divided into five age groups (10-24 years, n = 38; 25-39 years, n = 52; 40-54 years, n = 40; 55-69 years, n = 27; ≥ 70 years, n = 12)	Assessment of total protein content, peroxidase activity, malondialdehyde, and NO levels in UWS	No differences in salivary volume; decreased peroxidase activity and increased NO levels with aging	Suggests age-related oxidative stress in saliva independent of salivary volume; lacked direct hormonal profiling
Foglio-Bonda et al., 2019^37^	120 women (60 postmenopausal; 60 fertile)	UWS pH and flow rate; DMFT index	Lower salivary pH and reduced flow rate in menopausal group; similar DMFT index	Standardized UWS collection demonstrates modest but significant salivary hypofunction
Bosman et al., 2022^43^	50 healthy women (24 aged 22-45 years; 26 aged 55-92 years)	UWS; untargeted metabolomics via LC-MS	Age-associated shifts in salivary metabolomic profile	Most differentially expressed metabolites in older women were related to amino acid metabolism, the Krebs cycle, fatty acid metabolism, and nucleic acid synthesis
Soundarya et al., 2022^36^	60 women (20 menstruating, 20 premenopausal, 20 postmenopausal)	UWS; salivary estradiol (ELISA); salivary calcium; xerostomia and periodontal indices	Lower salivary estradiol and higher calcium levels in postmenopausal group; decreased estradiol associated with higher xerostomia and periodontal scores	Potential screening markers; standardization required
Divya Harika et al., 2024^26^	60 women (30 premenopausal; 30 postmenopausal)	UWS; taste thresholds; serum zinc levels	Postmenopausal women exhibited reduced sweet and bitter taste perception; no differences in salivary flow or zinc levels	Taste alterations may be multifactorial beyond zinc deficiency or flow reduction; neurosensory factors should be considered
de Moraes et al., 2024^27^	85 autopsied women (reproductive phase ≤ 44 years, n = 18; climacteric phase 45-55 years, n = 21; postmenopause ≥ 56 years, n = 46)	Morphological analysis of labial minor salivary glands	Aging associated with acinar fibrosis and atrophy, serous metaplasia, adipose replacement, and microthrombosis	Provides anatomical basis for salivary hypofunction; lacks correlation with clinical symptoms
García-Alfaro et al., 2025^42^	3,211 peri- (n = 1,170) and postmenopausal (n = 2,041) women aged 40-90 years	XI; oral health-related quality of life (OHIP-14)	Higher prevalence of xerostomia in peri- and postmenopausal women; associated with poorer quality of life	Supports routine xerostomia screening in menopause-focused care settings
Scardina & Messina, 2012^44^	27 premenopausal and 27 postmenopausal women	Oral microcirculation assessment (videocapillaroscopy); periodontal indices	Reduced periodontal capillary density, increased vascular tortuosity, and decreased loop diameter in postmenopausal women	Suggests vascular contribution to increased periodontal vulnerability
LaMonte et al., 2013^45^	1,025 postmenopausal women (5-year follow-up)	Longitudinal assessment of periodontal disease progression	Disease progression linked to systemic factors; overall no generalized progression observed; women with prior severe periodontitis experienced accelerated bone loss	High-quality cohort study; indicates risk stratification is necessary
Wactawski-Wende et al., 2020^14^	1,342 postmenopausal women at baseline; 1,026 (5-year follow-up); 518 (17-year follow-up)	Comprehensive oral examinations; bone density and body composition assessment; subgingival microbiome (16S sequencing)	Integrated oral and systemic findings in aging women	Landmark dataset informing oral-systemic risk models
Ahmad et al., 2024^39^	100 postmenopausal women	Full periodontal examination; bone densitometry	Age identified as a significant risk factor for bone loss in postmenopausal women	Supports risk-based periodontal screening; observational design limits causal inference
Yakar et al., 2024^46^	75 postmenopausal (38-65 years); 71 premenopausal (33-52 years)	Cytokine levels in serum and saliva (TNF-α, IL-6, IL-10, IL-7); BMI; Women's Health Questionnaire; periodontal assessment	No significant cytokine differences after age adjustment; fewer teeth in postmenopausal group; no differences in periodontal parameters	Age is a major confounder; salivary cytokines alone may not differentiate menopausal status; supports multifactorial etiology
Yakar et al., 2025^47^	77 premenopausal; 81 postmenopausal women	Checkerboard DNA-DNA hybridization (40 species) and 16S sequencing; serum estradiol (ELISA); modified SMDI	Despite more severe periodontitis, no menopause-related microbial dysbiosis observed; two species correlated with estradiol in premenopause	Microbial variation appears associated with disease severity rather than hormonal status; highlights systemic regulation of oral ecology

16S: 16S ribosomal RNA gene sequencing; BMI: body mass index; DMFT: Decayed, Missing, and Filled Teeth; ELISA: enzyme-linked immunosorbent assay; IL-6: interleukin 6; IL-7: interleukin 7; IL-10: interleukin 10; LC-MS: liquid chromatography-mass spectrometry; NO: nitric oxide; OHIP-14: Oral Health Impact Profile, 14-item version; SMDI: Subgingival Microbial Dysbiosis Index; TNF-α: tumor necrosis factor alpha; UWS: unstimulated whole saliva; XI: Xerostomia Inventory.

### Oral health, menopause, and cardiovascular disease

The decline in estrogen during menopause affects not only the oral cavity but also accelerates cardiovascular risk, which remains the leading cause of death in women. Women of reproductive age exhibit a lower incidence of cardiovascular disease compared with men; however, this difference diminishes after menopause, when cardiovascular risk increases substantially.^8,32^ The menopausal transition therefore represents a pivotal stage in women's cardiometabolic health.

Hypoestrogenism during menopause is associated with reduced NO bioavailability, a molecule with anti-inflammatory, antioxidant, and anti-atherogenic properties.^18^ At the same time, menopause is characterized by increased production of pro-inflammatory mediators, including tumor necrosis factor-α, interleukin-6, and C-reactive protein. These cytokines, including those originating from inflamed periodontal tissues, may enter systemic circulation and contribute to endothelial dysfunction, atherogenesis, and plaque instability, which are central mechanisms in cardiovascular pathology.

Within the cardiovascular system, menopause is associated with endothelial and vascular smooth muscle dysfunction as well as microvascular atherosclerosis. These alterations may lead to nonobstructive coronary artery disease driven by coronary microvascular dysfunction.^17^ In the United States, postmenopausal women with estrogen deficiency represent a substantial proportion of patients diagnosed with non-obstructive coronary artery disease.^33^

Paralleling the evidence of microvascular dysfunction in the cardiovascular system, Uhl et al. reported that several pathological conditions of the salivary glands are associated with inflammation-related microvascular disease.^34^ However, the inflammatory impact of menopause on salivary gland microvasculature and its consequences for salivary composition and flow remain insufficiently investigated. Some studies have suggested that microvascular thrombotic changes in salivary glands, combined with systemic effects of hypoestrogenism such as hypertension, may impair glandular parenchyma. This process may lead to glandular atrophy and the clinical manifestation of xerostomia.^16,27^

A growing body of evidence supports an interdependent relationship between menopause, periodontal disease, and cardiovascular risk. Estrogen exerts vasodilatory, anti-inflammatory, and antioxidant effects on the vascular endothelium, partly through maintenance of NO levels.^8^ Its decline during menopause has been associated with increased arterial stiffness, adverse lipid profile changes, and altered coagulation patterns, all of which contribute to increased cardiovascular risk.^35^

Simultaneously, hypoestrogenism promotes oral mucosal atrophy, salivary dysfunction, and alveolar bone resorption, creating conditions conducive to periodontal disease.^7,14,37^ These parallel mechanisms suggest that estrogen serves a central regulatory role in both oral and cardiovascular health. Additionally, changes in salivary pH have been associated with inflammatory status, oxidative stress, and NO metabolism, which may have systemic cardiovascular implications.^22,37^

Menopausal women demonstrate increased prevalence and severity of gingivitis and periodontitis. These changes may result from direct hormonal effects as well as systemic alterations, including reduced bone mineral density and dysregulated immune responses.^14,38^ Estrogen deficiency appears to amplify inflammatory responses to periodontal pathogens, increase connective tissue breakdown, and impair alveolar bone remodeling. These processes accelerate loss of periodontal attachment and may ultimately lead to tooth mobility and tooth loss if untreated.^14,39^

Postmenopausal osteoporosis may further exacerbate loss of alveolar bone, reinforcing a potential link between skeletal health, chronic inflammation, and cardiovascular disease.^1,39,40^ Chronic low-grade inflammation associated with periodontitis has been implicated in the development and progression of coronary artery disease, stroke, and peripheral arterial disease. This process is initiated by oral microbial dysbiosis and perpetuated by host immune responses.^1,39,41^

For clinicians, recognition of this triad, menopause, oral disease, and cardiovascular risk, should inform comprehensive history-taking and risk assessment. Periodontal status may serve as a marker of systemic inflammation and cardiovascular vulnerability. Conversely, unexplained oral symptoms in postmenopausal women may justify broader metabolic and vascular evaluation.^40^ Public health strategies targeting midlife women should integrate oral health promotion into cardiovascular prevention programs, particularly in aging and underserved populations.

Beyond inflammatory mechanisms, menopause-related oral and cardiovascular conditions share several modifiable risk factors, including smoking, diabetes mellitus, obesity, physical inactivity, and unhealthy dietary patterns. Psychosocial stress and sleep disturbances, both common during menopause, may further exacerbate systemic inflammation and reduce health-seeking behaviors, compounding the risk of both periodontal and cardiovascular disease.^1,14,42^

### Knowledge gaps and future directions

Despite increasing recognition of the relationship between menopause and oral health, substantial gaps remain in both scientific evidence and clinical implementation. These limitations hinder the development of standardized care pathways and evidence-based interventions tailored to menopausal women.

Menopause is a biopsychosocial transition that extends beyond physiological changes, often affecting mood, cognition, and emotional resilience. Oral manifestations occurring during this period may significantly influence self-perception, social participation, and overall quality of life. They may also contribute to psychological stress, anxiety, and depressive symptoms. Despite their impact, these oral complaints are frequently underreported by patients and underrecognized in both medical and dental practice.

Oral integrity is essential for mastication, swallowing, phonation, and nonverbal communication. Menopausal oral symptoms, particularly those related to mucosal atrophy, compromised dentition, or salivary hypofunction, may impair these fundamental functions. Eating and speaking difficulties in social contexts may lead to embarrassment and social withdrawal. Dissatisfaction with oral aesthetics, including tooth discoloration, tooth loss, and mucosal changes, may further negatively affect self-esteem and body image, which may already be vulnerable during this life stage.

Addressing these knowledge gaps requires a coordinated multidisciplinary research agenda, supportive health policies, and a transition toward patient-centered, life-course-based care models. The longstanding separation between dental and medical care remains a major barrier to comprehensive management. Although oral symptoms are common during menopause, few women receive structured dental evaluation as part of routine gynecologic, cardiologic, or primary care visits. Conversely, dental professionals may lack standardized protocols to screen for systemic conditions such as osteoporosis, cardiovascular disease, or metabolic syndrome that influence oral health.

Emerging technologies, including salivary diagnostics, microbiome sequencing, and digital periodontal assessment, offer promising tools for earlier and more precise identification of menopause-related oral conditions. Further research is required to validate these approaches in diverse menopausal populations and to evaluate their clinical applicability in routine practice.

Improved understanding of the relationship between oral disturbances and cardiovascular symptoms, such as angina or ischemic heart changes, may also contribute to more comprehensive cardiovascular risk stratification in menopausal women.

## Data Availability

The underlying content of the research text is contained within the manuscript.
